# Methodology for dissolution of sediment and calcareous deposits for paleontological specimen collection and identification

**DOI:** 10.1016/j.mex.2022.101816

**Published:** 2022-08-17

**Authors:** Jack Schultz, Griffin Scheurer, Lydia Tackett, Dianna Berry

**Affiliations:** a Science Research Program, Westhampton Beach High School, 49 Lilac Road Westhampton Beach, NY 11942, United States; b Department of Earth, Environmental, and Geospatial Science, North Dakota State University, Fargo, ND 58102, United States

**Keywords:** Paleontology, Sample dissolution, Lance creek, Extinction event, Small macrofossils, Biodiversity

## Abstract

The Lance Creek Formation (Late Cretaceous) is significant in the study of late dinosaurs and is a diverse formation containing both terrestrial and aquatic macrofossils. To study this important ecosystem, all of the fossils present must be uncovered and examined from Lance Creek sedimentary rocks, which exhibit variable degrees of lithification. In order to liberate the fossils, a dissolution methodology was designed to determine which solution was most effective at uncovering specimens and dissolving/disaggregating sediment. Different solutions were tested including water, a 50% Calgon© solution, and a 5% acetic acid solution. The control was a sedimentary rock sample not subjected to any solutions prior to rinsing and sieving. A small-scale dissolution (10 g of loose sediment) was performed using each solution and examined for fossils. Acetic acid was deemed the most effective solution for the dissolution of dense sandstones, and indurated sediment from the Lance Creek Formation. Large-scale disaggregation (800 g of consolidated sedimentary rock) yielded abundant terrestrial, fluvial, and marine macrofossils. Macrofossil disaggregation using these methods has the potential to yield a more diverse assemblage of contemporaneous fossils than macrofossils alone, and can therefore provide substantial insight into ecological reconstructions.

• A small-scale study was done to determine which solution was the most efficient at dissolution of sediment. Acetic acid was deemed the most effective.
• A large-scale experiment was done on dense sandstones using 5% acetic acid and a shaking incubator.
• A large-scale experiment of indurated sediment was performed using 5% acetic acid.

## Highlights

• A small-scale study was done to determine which solution was the most efficient at dissolution of sediment. Acetic acid was deemed the most effective.
• A large-scale experiment was done on dense sandstones using 5% acetic acid and a shaking incubator.
• A large-scale experiment of indurated sediment was performed using 5% acetic acid.

## Graphical Abstract

**Figure:**
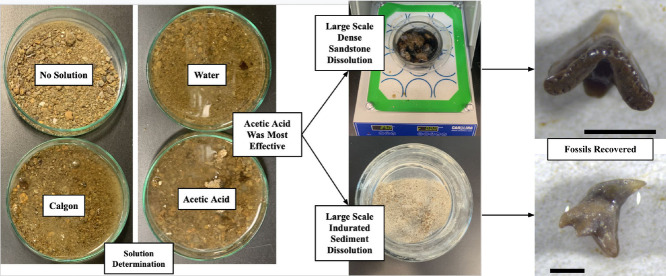

Specifications table

**Table:** 

Subject area:	Earth and Planetary Sciences
More specific subject area:	Paleontology
Name of your method:	The dissolution of sediment for small macrofossil collection and identification.
Name and reference of original method:	Jeppsson, L., Anehus, R., & Fredholm, D. (1999). The optimal acetate buffered acetic acid technique for extracting phosphatic fossils. *Journal of Paleontology, 73*(5), 964-972. doi:10.1017/S0022336000040798 Cureton, J. C., Lewis, P. J., & Thies, M. L. (2010). Evaluating Acetic Acid for Removing Microvertebrate Fossils from Cave Breccia. *Botswana Notes and Records, 42*, 172–178. Doi: http://www.jstor.org/stable/23237982
Resource availability:	n/a all resources are conventionally available.

## Methods

The methods explored here were developed because dissolution and disaggregation minimize the amount of sediment needed to be examined and increase the visual quality of smaller macrofossils. To decrease the amount of sediment to examine, and reduce collection bias for vertebrate fossils, an experiment with multiple dissolving solutions was completed and is described below. Calgon and acetic acid were chosen because of previous small macrofossil extraction studies, which showed promising results [1,2]. Calgon and acetic acid were specifically chosen since these materials are efficient in disaggregating and dissolving the Lance Creek Formation lithology respectively. The most common lithologies present in the Lance Creek are calcareous, medium to fine-grained sandstones, and light to dark gray, fissile, poorly indurated siltstone [3]. These sections were deposited in inland flood basins with expansive swamps, and a lagging northeastward discharging drainage system, overlaying the emerging seafloor [4]. Lance Creek deposits exhibit variable levels of carbonate content, observed both as calcareous biogenic grains (mollusk shells) and cements. In the process described here, liberated sediments smaller than approximately 250 microns were not examined. If one aspires to look at samples less than 250 microns, they can use a smaller pore size for the rinsing process.

Prior to the start of this dissolution experiment, a sample batch of sediment was examined to see if any of the smaller macrofossils were present and to characterize their condition without treatment by acids or surfactants.

### Sample collection

**Fig. 1:**
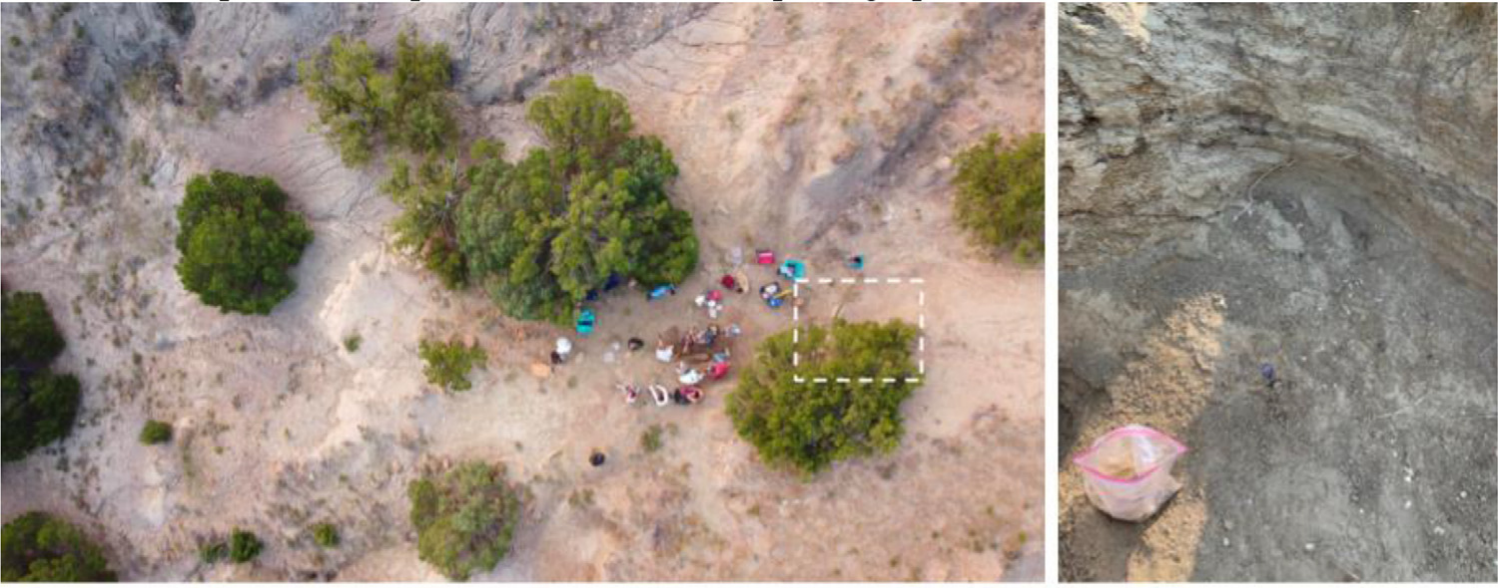
Left image is an aerial photograph of the sampled Lance Creek outcrop at Zerbst Ranch. General collection area outlined in dotted white box. Right image is the specific collection site along with sample in the Ziploc^Ⓡ^ bag. Ziploc bag is 26.8 cm in diameter. The samples were collected in summer of 2021 and excavation of an edmontosaurus tibia was taking place.

Two different types of material were collected for this study: indurated sediment and dense cemented sandstone. These materials were classified by indurated sediment being loose sediment which was scooped off the surface, while the dense cemented sandstone was harvested using a hammer and chisel. Large hand samples of fossiliferous medium to fine-grained sandstone of the Lance Creek were collected from the Zerbst Ranch (Fig. 1), ranging in weight from 800 g to 1.3 kg, with the largest piece being 18 cm long. Loose sediments were also collected from the same outcropping section and subjected to the same treatments as more lithified material, about 2.8 kg of sediment. The samples were placed into gallon Ziploc^Ⓡ^ bags and shipped to the research lab at Westhampton Beach High School (New York). Images of all samples were taken using an Excelis Accu-Scope microscope camera to view and photograph the small macrofossils.

### Comparative disaggregation methods for smaller samples of lance creek sandstones

To determine which solution was most effective for total weight reduction, an experiment was performed comparing the effectiveness of 5% acetic acid (vinegar), 50% Calgon© solution, water, and no solution. Water was used as a positive control, while no solution was used as a negative control. Ten grams of sediment were placed in a 10 cm diameter glass Petri dish, and 300 ml of the given solution was added to the dish. The solutions were incubated for 3 h at room temperature (19°C) and then were emptied into a 254 µm sieve and rinsed with warm water. This allowed the dissolved clay to separate from the sample and rinse off any remaining solution. Samples were then placed in a cabinet incubator for 24 h at 37°C to allow all of the water to evaporate. At the end of the 24 h incubations, the sediment was weighed. The mass of remaining material from each solution was compared to determine the effectiveness of each solution. The four treatments were performed in triplicate, and the data was compiled, so the average mass losses can be seen in the method validations.

### Acid-dissolution of larger samples of cemented lance creek sandstones

**Fig. 2:**
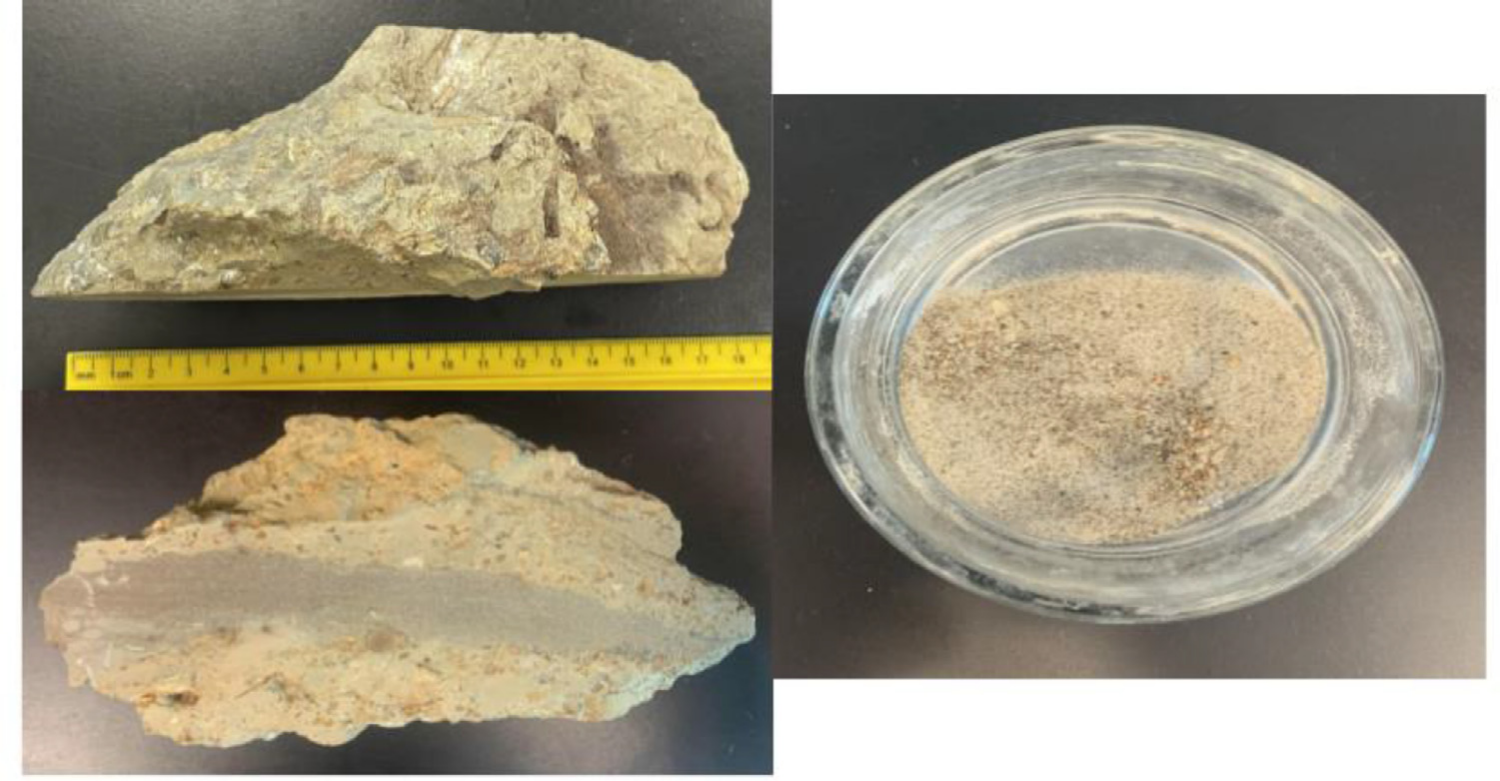
(Left) Cemented Lance Creek sandstone prior to going through the dissolution process. (Right) Indurated loose sediment prior to going through the dissolution process.

Lance Creek Formation sedimentary rocks exhibited variable degrees of lithification, and we sought to determine the effectiveness of acid dissolution on larger samples. To dissolve well-cemented dense sandstones (Fig. 2), the fossiliferous sandstones were first manually disaggregated to form fragments ranging from 1 to 5 cm and were added to a 0.4 m^3^ Pyrex dish. Acetic acid (5% distilled vinegar) was then added until the sample was fully submerged. The Pyrex dish was covered with a glass petri dish to prevent evaporation. The rock was then allowed to incubate for 48 hurs at room temperature (19°C). After 48 h, the buffered acid was decanted from the bowl. The samples were slightly softened after this initial treatment and could be disaggregated further by prodding with a plastic pipette using light pressure. If a piece would not break, it was left in the dish unbroken. After most of the chunks were fractured into smaller pieces, 30 ml of fresh acetic acid solution were added to submerge the rock fragments. The dish was placed on a shaking incubator (Carolina Incu-Shaker mini) at 22°C and 110 rpm until the sediments were completely disaggregated for up to 48 h. This allowed the sample to be agitated constantly, speeding up the dissolution process. During this period, the acetic acid was replaced every 24-48 h, and the sediment aggregates were poked with plastic pipettes. This process was repeated until nearly all of the rock separated.

### Acid dissolution of larger samples of indurated, loose lance creek sediment

Indurated sediments of Lance Creek sandstone (30 g) were combined with 90 ml of 5% acetic acid. The sediment and solution were incubated for three hours in a covered Pyrex dish at room temperature (19°C). The sediment was then rinsed with water in the 254 µm sieve and dried in the incubator at 29°C for 12-18 h. The sediment was then sifted through a progressive series of sieves (12700 µm, 6350 µm, 3175 µm, 2116 µm, 1270 µm, 838 µm, 508 µm, 355 µm, and 254 µm), allowing collection bias to be decreased. Each size fraction was then analysed by light microscopy, and fossils were documented through measurements and imaging. This experiment was repeated four times for a total of 120 g of sediment, and all sediment residues were examined in their entirety.

## Method validation

### Solutions

**Fig. 3:**
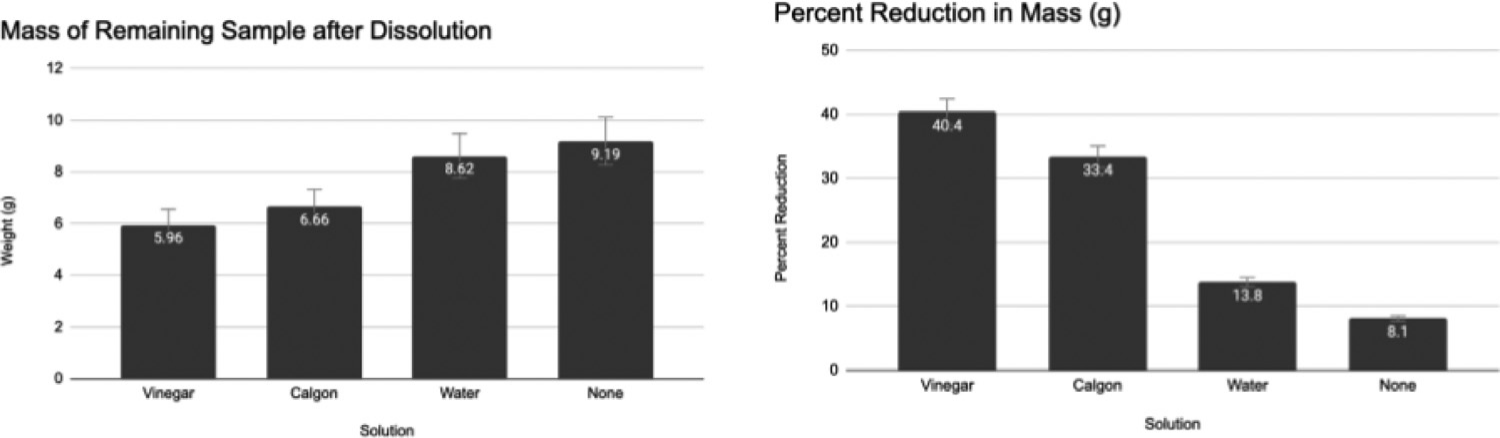
The final average weight of the samples and the percent weight loss after going through the dissolving process.

**Fig. 4:**
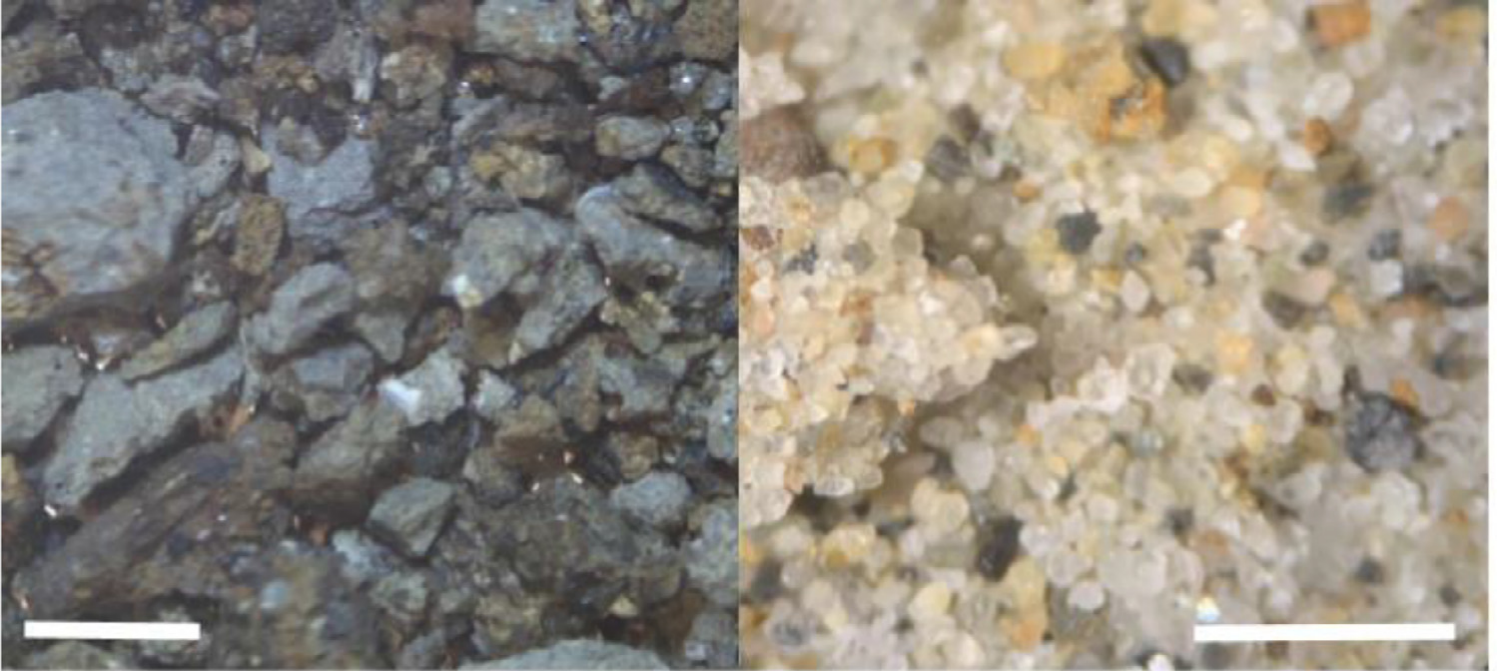
Sediment before being dissolved in acetic acid (left), and sediment after the acetic acid soak (right). Scale bar is 1 mm.

**Fig. 5:**
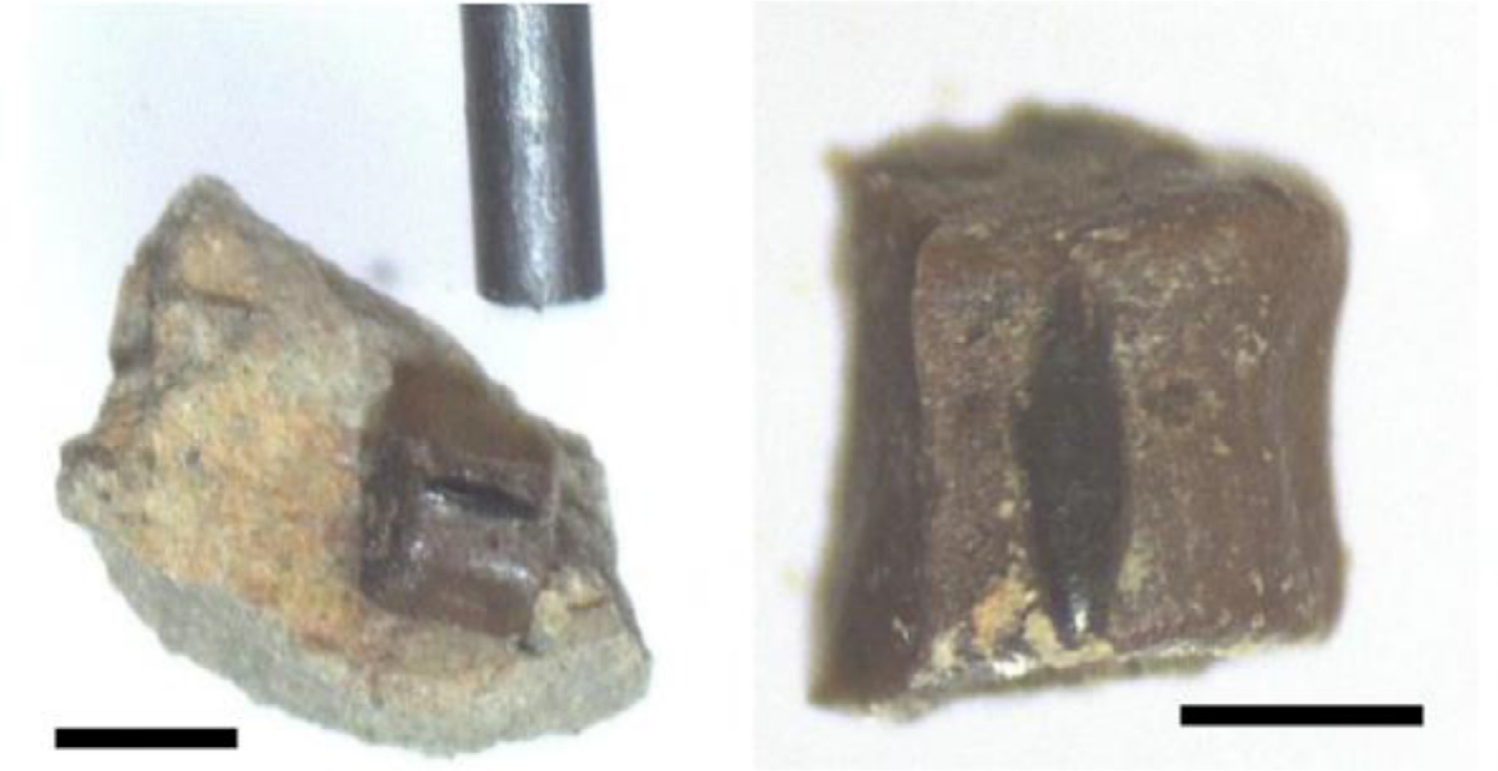
The left image is a gar caudal scale *in situ* before being dissolved in acetic acid. The right image is the same caudal scale after it had gone through the acetic acid procedures. The acetic acid removed the clay from the sample and became slightly polished. Scale bar on left is 1 mm, on right is 0.5 mm.

For loose sediments, the data showed that the 5% acetic acid solution had over a 40% reduction in mass, while the sample soaked and rinsed in water had only a mass loss of approximately 14% (Fig. 3), suggesting that the acetic acid solution was the most effective treatment in terms of removing smaller (<254 microns) matrix. Loose sediment (Fig. 4) and macrofossils (Fig. 5) were less encrusted by cements following the acetic acid treatment.

### Fossils

**Fig. 6:**
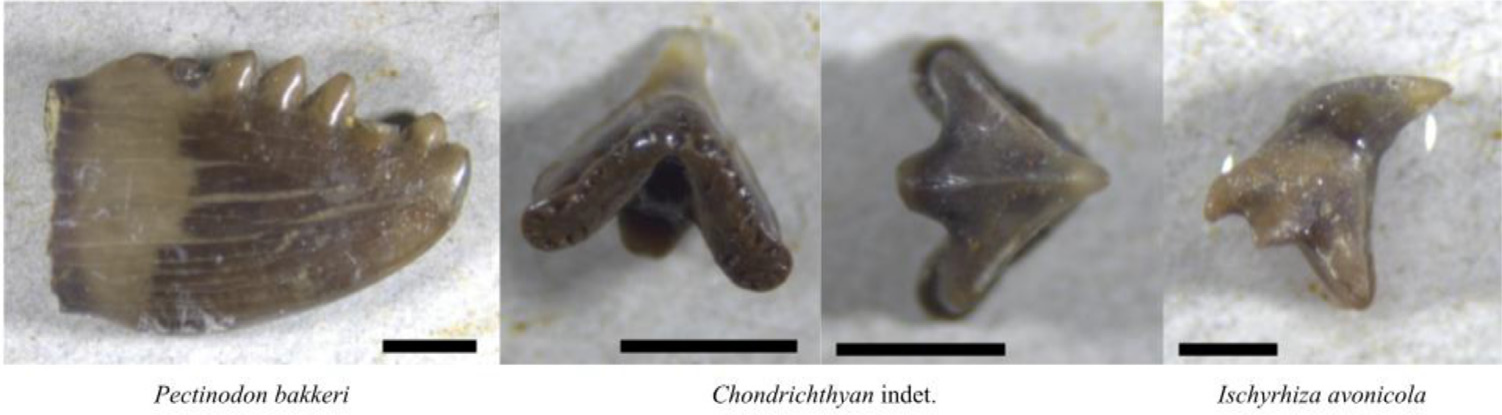
Examples of fossils collected from sediment after the dissolution process. Scale bars are 1 mm.

Throughout the disaggregation-dissolution process, a plethora of vertebrate macrofossils were collected, and a subset is illustrated herein, including a variety of teeth (Fig. 6). A further larger-scale study will be performed in the future, including an in-depth analysis of the teeth, and the Lance Creek biodiversity as a whole. Four samples were imaged using a Zeiss Discovery V.12 SteREO microscope with AxioCam ICc 5 camera and Z-stacking software at North Dakota State University.

## Discussion

The methodology described here for dense sandstone and indurated sediment dissolution will enable a more efficient process for identifying macrofossils by reducing the amount of sediment needed to be sorted through and allowing samples to be identified more easily. If more macrofossils are collected, there may be a decrease in collection bias due to the increase in the range of sample collection size. More comprehensive faunal lists enable better palaeoecological analysis, and likely result in new taxonomic descriptions of previously unrecognized species. This methodology will also enable more accurate imaging and identification of specimens since they will appear cleaner and polished, allowing for more distinct features to be observed. Identifying more specimens will also lead to a more accurate understanding of the biodiversity within late Cretaceous microsites and potentially give a better understanding of the ecosystem and rate of deposition in that area.

### Implications

The designed methodology may not be applicable for all lithological sections or specimen recoveries. This method was designed around a known chemical and mineralogical composition of the fossils. If the composition of the fossils is calcareous, then the method will be destructive to specimen collection. The methodology may not be applicable for some rock types where the cement does not react to acetic acid., and a different solution would need to be used. The methodology designed works best for phosphate fossils encased in rock bound by calcareous cement.

### Educational outcomes

This methodology was designed and tested by high school students under the supervision of experienced researchers and experts in the field of paleontology. This methodology allowed students to gain a more comprehensive understanding of designing a methodology, constructing their findings in a well written report, and understanding the publication process.

## CRediT authorship contribution statement

**Jack Schultz:** Methodology, Conceptualization, Data curation, Writing – original draft, Writing – review & editing. **Griffin Scheurer:** Methodology, Conceptualization, Writing – review & editing. **Lydia Tackett:** Validation, Resources, Writing – review & editing, Data curation. **Dianna Berry:** Validation, Resources, Supervision, Writing – review & editing.

## Data Availability

Data will be made available on request.
